# The issue of interconnections and mutual influence of digital transformation in international trade: How digitization affects customer satisfaction?

**DOI:** 10.1016/j.heliyon.2024.e36854

**Published:** 2024-08-28

**Authors:** Guangying Wang

**Affiliations:** a School of International Business, Qingdao Huanghai University, Qingdao, China; b Department of International Commerce & Business, Konkuk University, Seoul, South Korea

**Keywords:** Digitization of international trade, Customer satisfaction, Digital innovations, Expectancy-confirmation theory, SERVQUAL

## Abstract

This research aims to investigate the relationships and mutual influence between the digitalization of international trade and customer satisfaction in the context of the post-COVID-19 economy. To achieve this objective, a comprehensive research project has been developed, involving a quantitative approach that combines the advantages of experiments and surveys. Customer satisfaction was assessed based on key service quality parameters in the SERVQUAL model. The study involved 4 stores operating on the AliExpress international trading platform. The sample included a total of 3796 customers. The research findings confirmed the impact of digitalization on customer satisfaction in international trading operations across nearly all dimensions of the SERVQUAL model. The implementation of basic digital innovations substantially enhanced reliability, order processing and delivery speed, product packaging, and the process of selecting and ordering goods, as well as the regularity of notifications and data security. However, the introduction of a chatbot significantly reduced customer satisfaction with the provision of additional information, leading to a decrease in customer empathy. The academic contribution of this study lies in its ability to complement and expand the scientific understanding of the impact of digitalization on customer satisfaction in international trade in the post-COVID-19 era. The study demonstrated that not all digital innovations are perceived equally positively by customers and do not necessarily lead to increased satisfaction. Accordingly, the practical outcome of this research is the empirical confirmation of the need to test customer satisfaction when implementing digital innovations.

## 1 Introduction

In the contemporary global economy, digitization emerges as a pivotal factor influencing business development [1], thereby driving research interest in investigating such influence in service and trade operations [2], including international trade activities and their implementation [3]. Digital transformation contributes to enhancing the efficiency of international trade operations. This process facilitates document flow, customs clearance, logistics, and payment transactions by reducing employee errors and optimizing supply chain management [4]. The economic repercussions of the COVID-19 pandemic have fostered the active digitization of international trade, accelerating the transition of trade organizations to a digital mode of operation [5].

In the context of the global economy, the increasing pace of digitalization is leading to rapid changes in the business landscape. Therefore, it is essential to study the impact of digital transformation on international trade, prompting scientific research to pay special attention to these issues [6]. Some studies investigate specific research direction related to the impact of digitalization on customer satisfaction [7]. In particular, researchers emphasize that in the current conditions, marketers need to deepen their understanding of consumer segments based on the behavior associated with the pandemic [8]. The issue of customer satisfaction has also been in the focus of attention among theorists and practitioners for a long time. Previous studies have assessed the relationship between service quality and customer loyalty and satisfaction with cloud services [9]; developed digital tools to assist in extracting information from customer reviews in order to further enhance customer satisfaction [10]; determined the correlations between the indicators of service quality and customer satisfaction [11]. A significant number of modern studies investigate the interaction between cognitive structures, customer engagement, and behavioral choice of companies in the context of customer satisfaction during COVID-19 and increased digitalization of trade [12,13]. Researchers have analyzed the correlation between consumer sentiment and behavioral intentions in decision-making in the context of previous customer satisfaction experiences [[14], [15], [16]]; developed targeted technology adoption models [17]; and presented expectation confirmation theories for evaluating customer satisfaction with digital platforms and in the field of digital solutions [18]. Some studies demonstrate evidence-based data on the impact of digital commerce during COVID-19 on the quality of customer service for large retailers [19].

Thus, a significant amount of research has been conducted to address certain aspects of the impact of digitalization on services, trade, and sales. Nevertheless, there is still a lack of data on the relationship between the digitalization of international trade and customer satisfaction. Particularly, the priorities that shape consumer behavior, expectations and, consequently, shopping satisfaction deserve special consideration. Researchers highlight the need for developing recommendations to better understand consumer demand, formulate marketing strategies [20], comprehend changes in consumer behavior regarding online shopping habits [21], and design strategies for managing customer relationships in the digital age [22].

This study aims to fill the current research gaps and create an empirical basis for understanding the impact of digitalization on customer satisfaction in international trade in the post-COVID-19 economy. Accordingly, the research questions are as follows: How does digitalization affect the level of customer satisfaction in international trade? What impact do specific elements of digitalization have on customer satisfaction in the post-COVID-19 economic environment? To achieve the research goal and answer the formulated questions, it is necessary to accomplish the following objectives: to summarize the main theories for assessing customer satisfaction; to assess the overall impact of digitalization on customer satisfaction in modern international trade; to assess the positive and negative impact of specific elements of digitalization on customer satisfaction with shopping on online platforms.

## 2 Literature review

### 2.1 Key theories of customer satisfaction

The issue of assessing customer satisfaction and the factors influencing customer satisfaction, as mentioned earlier, has long been the focus of researchers' attention. In general, a chronological analysis of the main theories of customer satisfaction can be summarized and presented in a table (Table 1).

**Table 1:** Chronological analysis of key theories of customer satisfaction.

Title	First Mentions	Most Notable Followers
Dissonance Theory	1957	[[20], [21], [22], [23], [24], [25], [26], [27]]
Assimilation-Contrast Theory	1961	[[28], [29], [30], [31], [32], [33], [34]]
Adaptation Level Theory	1964	[[35], [36], [37], [38], [39], [40], [41]]
Expectancy-Confirmation Theory	1977	[[42], [43], [44], [45], [46]]
Value-Percept Theory	1983	[[47], [48], [49], [50]]
Evaluative Congruity Theory	1984	[[51], [52], [53], [54]]

There are several theoretical approaches to studying customer satisfaction. The earliest theory of customer satisfaction in contemporary academic research is the dissonance theory [23]. This theory was first proposed by Festinger [24] and later significantly refined by Cardozo [25], Cardozo and Cagley [26]. According to the dissonance theory, an individual faced with a discrepancy between expectations and experiences (for example, receiving a low-value product with high expectations) experiences cognitive dissonance [25], which has subsequently been characterized as psychological discomfort [27]. To alleviate such discomfort, individuals may alter their perceptions of the product, reassess their experience, and even assert that the discrepancy is a positive aspect of aligning expectations with reality. The theory has gained popularity in social research due to its few principles (logical inconsistencies) that can explain the complex process of dissonance. However, the lack of arguments has raised concerns about the possibility that interfering variables (other psychological and cultural factors) are overlooked in the theory [28]. It is also argued that dissonance is difficult to reproduce in a real experiment [29].

The theory of assimilation and contrast asserts that the discrepancy between consumer expectations and the actual characteristics of the product intensifies the perception of discrepancy [30]. When the actual value of a product does not meet expectations, consumers tend to exaggerate the difference between expected and actual outcomes – both in positive and negative discrepancies. It is noted that the theory of assimilation and contrast (as well as the above-mentioned dissonance theory) has been studied in terms of its applicability in practice only in experimental settings with tightly controlled customer satisfaction [25,31,32]. Accordingly, its applicability in real-world situations is subject to skepticism [23].

Adaptation Level Theory posits that an individual's level of satisfaction depends on how their current situation corresponds to or deviates from established standards and expectations. According to this theory, an individual's perceptions of their expectations and desires are shaped by multiple factors simultaneously, including (1) prior consumption experiences of similar products; (2) situationally induced expectations created by the advertising efforts of the seller and/or manufacturer; (3) the experiences of other consumers serving as referent individuals in this case [33]. This theory complements the basic principles of studying consumer behavior and satisfaction. However, its practical applicability did not become widely accepted until it was supported by the postulates of motivational psychology [34].

The Value-Percept Theory analyzes the impact of personal values and sociocultural prescriptions on the formation of customer satisfaction. Customer satisfaction, according to this theory, is seen as an emotional response that occurs during cognitive evaluation. The latter is the process of a comparison between the product or service being offered and an individual's own values, needs, and desires [35]. The theory asserts that the level of satisfaction depends on the alignment of the offered product or service with the individual's personal values and conformity to societal prescriptions and expectations. Despite its important contribution to the development of approaches to assessing customer satisfaction, this theory somewhat neglects external factors and individual differences in needs and preferences. Moreover, it pays insufficient attention to situational factors (for example, a social or economic context), which can have a significant impact on satisfaction [36].

The Evaluative Congruity Theory conceptualizes customer satisfaction as a function of evaluative congruity. The theory defines cognitive comparison as a process in which perception is juxtaposed with referential knowledge to assess a stimulus or action [37]. In terms of evaluative congruence, customer satisfaction is an emotional state that motivates the evaluation of alternative courses of action to alleviate existing dissatisfaction and/or prevent similar states in the future [37]. This theory distinguishes three states of congruence: (1) negative incongruence, arising from dissatisfaction; (2) congruence, characterized by a neutral evaluative state or satisfaction; (3) positive incongruence, characterized by satisfaction [37]. In recent decades, this theory has been adapted to new aspects of studying customer satisfaction. However, in the era of digital transformation and interaction with customers in digital formats, it may require further refinement in order to be effectively applied for assessing customer satisfaction expectations and needs [38].

One of the most widely recognized theories is the Expectation Theory (also known as (Expectancy-Disconfirmation Theory) developed by Oliver [39]. According to the theory, customer satisfaction depends on how well a product or service aligns with their expectations. If expectations are confirmed, the customer experiences satisfaction, whereas a misalignment between expectations and experience leads to dissonance, diminishing the level of satisfaction. Building upon the postulates of the Expectation Theory, several models have been developed to understand and enhance customer satisfaction, including the SERVQUAL model [40], which assesses service quality based on customer perceptions and expectations. The SERVQUAL model comprises five fundamental dimensions of service quality: (1) reliability; (2) responsiveness; (3) assurance; (4) tangibles; and (5) empathy [40]. A comparison of customer perceptions and expectations for each parameter enables the identification of gaps in service provision and areas for improvement. Consequently, it is possible to assess how well service quality aligns with customer expectations in specific aspects of performance. Naturally, the model has some limitations related to the evaluation of digital trading instruments. For example, it does not take into account that consumers may have varying levels of satisfaction with different online platforms, and their expectations may vary depending on markets. In addition, the model relies solely on self-reported customer expectations (the Likert scale is used to measure the gap between expectations and perception) [41]. Nevertheless, the model can be applied to study customer satisfaction (in particular, the gap between consumers’ expectations and perceptions of service innovations) in the context of one online platform and one market (for instance, consumer goods, education, banking, travel services, etc.) [42]. The SERVQUAL model also has advantages that can facilitate the evaluation of customer satisfaction in the conditions of digitalization. Thus, it is widely used to measure the quality of service, providing a basis for assessing the level of satisfaction based on perceptions and expectations regarding the above key parameters [43]. Additionally, the elements of the SERVQUAL model for assessing customer satisfaction can later be utilized to establish service quality standards [44], which is essential for online trading, including in international markets.

### 2.2 Studying customer satisfaction in the context of digitization

Despite the close attention of academic researchers worldwide, the concept of digitization currently lacks a unified definition [45,46]. Digitization is understood as (1) "the process of digital company growth through the proliferation of digital technologies” [47]; (2) "the culmination of the information revolution, when the digital economy becomes the driving force of market agents in expanding access to resources and markets” [48]; (3) "the saturation of the physical world with electronic and digital devices, means, systems, and the establishment of electronic communication exchange between them, effectively ensuring the holistic interaction of the virtual and physical, thereby creating a cyber-physical world” [49]. In international trade, digitization primarily refers to the process of integrating digital technologies and innovations to enhance and optimize various aspects of trading activities, such as marketing, sales, logistics, and customer interaction. This process is aimed at creating a sustainable, efficient, automated, and convenient trading environment for enterprises and consumers. Activities promoting the digitization of international trade may involve the implementation of a virtual fitting room (at the purchase decision stage), additional forms of online payments, including virtual payment methods (at the payment stage), order tracking (at the delivery stage), customer support chatbots on messaging platforms, and so forth. Thus, **Hypothesis 1** can be formulated as follows: Digitization significantly influences the level of customer satisfaction in international trade under economic conditions post-COVID.

It is noteworthy that the growing demand from the real sector of the economy is fueled by intensified competition amidst overall economic turbulence. This has led to increased interest not only in issues related to customer satisfaction in the digital economy but also in specific aspects of its impact on satisfaction. Dost et al. [50] identify key factors influencing consumer decisions regarding digital purchases, such as (1) confidence, (2) convenience, (3) choice variety, (4) confidentiality, and (5) time savings. Grieve [51] emphasizes the importance of integrating customer insights into the development of customer-oriented business models to enhance consumer value. Contemporary scholarly thought provides empirical evidence that digitization in international trade not only promotes the advancement of differentiated products, but also creates diversified customer experiences [52]. Moreover, this opens up new forms of real-time customer interaction and more efficient service methods [53]. In the modern world, the unique and positive experience of digital purchases maximizes efficiency by prompting trading organizations to deepen their interaction with customers, promptly respond to their requests, and prevent problems. However, digitalization can also create new issues related to the formation of different conditions for making purchases, which customers need to get accustomed to. These may include discomfort due to the lack of physical contact with a product and disparities between expectations and the actual appearance of the product [54]. Hence, **Hypothesis 2** is formulated as follows: Specific elements of digitization have both positive and negative effects on customer satisfaction in economic conditions after COVID-19. This research aims to identify the interconnections and mutual influences of the digitization of international trade on customer satisfaction in the post-COVID economic environment.

## 3 Methods and materials

### 3.1 Sample

For the experiment, four stores engaged in international trade through the AliExpress platform were selected. Two of the chosen stores specialized in a wide range of home goods, one store offered a diverse assortment of hobby and leisure products, and the remaining store was associated with the fashion industry. The criteria for the inclusion of AliExpress in the experiment was its ability to present a representative context for studying the impact of digitalization on customer satisfaction. This trading platform provided a diverse sample in terms of demographic characteristics. The platform actively implements digital tools in customer service, as well as features stores with various consumer goods. The main arguments for the use of this trading platform are: (1) its scale, as AliExpress is a global virtual marketplace delivering goods to 254 countries worldwide; (2) a significant number of users (over 431 million visits a month and over 50 million active users in 2023); (3) the absence of domestic sales in China, as AliExpress is exclusively intended for international trade (AliExpress, a subsidiary of Alibaba, specializes only in international trade and is the leading platform for cross-border B2C trading); (4) the success of sellers who are retailers and supply products from factories for export depends (among other things) on the effectiveness of consumer service infrastructure implemented on the AliExpress platform. The use of the AliExpress platform determined the selection of innovations (additional options, including “AliExpress Fast Shipping,” “AliExpress Combined Fast Shipping,” and “Additional Delivery Time Insurance”). The experiment included stores on the AliExpress platform that had not implemented these additional features. The number of stores was limited to four, as this provided an optimal balance between the duration of the study and the number of respondents while remaining within the budget constraints of this research.

The sample of participants in this study consisted of AliExpress customers with accounts on the platform who made purchases in the selected stores during the research period. Invitations were distributed through newsletters to the store's subscribers, official store channels, and thematic communities on social networks. Respondents were invited in two phases: the first phase included 1807 respondents, and the second phase included 1989 respondents. The first phase involved participants from 67 countries, the second covered 73 countries. The majority of respondents were men (about 62 %). The most represented age ranges were 36–45 years (about 23 %) and 46–55 years (about 28 %). To assess the impact of innovations related to customer support organization on customer satisfaction, specific respondents from the two phases were invited (425 respondents of the first phase and 459 respondents of the second phase). These respondents made a purchase and contacted the customer support of the stores and/or the trading platform during the research period. The survey was conducted from September 25 to November 25, 2023.

### 3.2 Study design

**Fig. 1:**
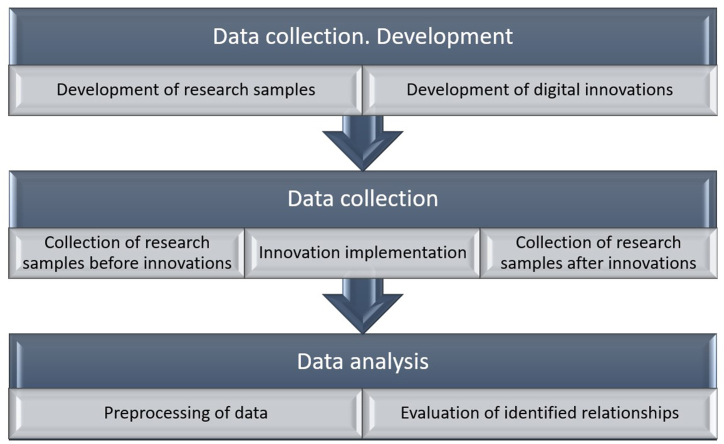
Methodological design of the study.

To achieve the ultimate goal of the research, a comprehensive research project was developed and implemented. The project involved the utilization of a quantitative approach (Fig. 1).

The methodological design of the study combines the advantages of both experimental and survey methods. At the preliminary stage, an algorithm for the survey process was developed, which included setting the purpose of the survey; developing questionnaires; verifying the validity of the developed questionnaires. The first stage of the study focused on the development of key data collection measures and includes two consecutive stages: the creation of research samples and the development of digital innovations used in the experiment. Digital innovations were introduced during the experiment to identify the impact of specific elements of digitalization on customer satisfaction. Next, a survey was conducted and its results were processed. The second stage of the study was the sequential collection of research samples, from digitization to the introduction of digital innovations and post-digitization data collection. The third stage involved analyzing the collected data (the survey results) and verifying the fulfillment of the research goal. This stage included systematic preliminary data processing and evaluating the identified relationships in order to test hypotheses H1 and H2. Thereafter, the theoretical implications of the study and the practical implications for management decision-making were formulated.

### 3.3 Methods and research tools

The analysis employed a combination of general scientific and specialized methods. Content analysis and the chronological method were utilized in analyzing theoretical sources. These approaches were used to scrutinize the arrays of publications and subsequently interpret the research findings in the field of theories, factors, and approaches related to customer satisfaction in the context of digitalization [24,26,30,33,37,40].

Brainstorming, the Delphi method, and analysis-synthesis methods were employed in developing the research methodology. These methods enabled a collective search for solutions in order to develop an approach to the systematic and structured collection of expert opinions. As a result, it was possible to gain insight into the connections between the components of customer satisfaction in terms of digitalization [50,53,54]. Experimental and online survey methods were used for data collection. During the data processing stage, the encryption method was utilized to compactly represent extensive information arrays.

The questionnaire design methodology rests on the advantages of the SERVQUAL model [52,55] as well as insights from prior research [12,13,56]. Customer satisfaction was assessed according to key service quality parameters in the SERVQUAL model. These parameters include: reliability (in this case, the ability to reliably and accurately perform customer service of the store on AliExpress); responsiveness (willingness to provide customers with fast trading operation service and assistance); completeness (intuitive process of choosing and making an order, reliable packaging, and fast delivery), confidence (confidence in the security of data during purchase and timely notification of the order status); empathy (a caring attitude towards customers and an personalized approach to each of them) [13,52,56]. The questionnaire is presented in Appendix A.

The survey was an appropriate tool for collecting data from the desired population. This method allowed for focusing on the formulated objectives and goals of the study. To assess customer satisfaction, a survey questionnaire incorporated: (1) open-ended questions; (2) closed-ended questions with single-choice responses; (3) open-ended and closed-ended questions with single-choice responses; and (4) Likert scale questions. Closed-ended questions with single-choice responses were predominantly used in constructing the respondent profile, while a 5-point Likert scale (agreement scale) was directly employed to evaluate customer satisfaction. The Likert scale ranged as follows: strongly disagree – 1 point; somewhat disagree – 2 points; neutral – 3 points; somewhat agree – 4 points; strongly agree – 5 points. Reliability was assessed using tau-equivalent reliability. The Cronbach's α coefficient of 0.88 indicates the stability of the scale and its compliance with the research objectives. According to the AVE criterion, convergent validity is sufficient (not lower than the established threshold value of 0.5). The acceptability, normality, and reliability of the sample are confirmed by the total correlation coefficient (it exceeds 0.7 for all questions).

To assess the normality and reliability of the sample, the study employed the «Item-total correlation » method [10]. The Pearson correlation coefficient for determining the normality and acceptability of questions using the «Item-total correlation » method was calculated according to the formula:

$$r_{xy}=\frac{\sum_{i=1}^{m}\left(x_{i}-\bar{x}\right)\left(y_{i}-\bar{y}\right)}{\sqrt{\sum_{i=1}^{m}{\left(x_{i}-\bar{x}\right)}^{2}\sum_{i=1}^{m}{\left(y_{i}-\bar{y}\right)}^{2}}}=\frac{cov\left(x,y\right)}{\sqrt{s_{x}^{2}s_{y}^{2}}}$$

### 3.4 Ethical issues

Participation in the study was non-personalized, thus not requiring additional consent for the processing of personal data. However, the survey questionnaire was only sent after the respondent had activated their consent for the processing of the information they provided in the questionnaire. The anonymity of the stores participating in the experiment was ensured.

### 3.5 Methodological limitations

The study is limited by methodological tools – the use of the SERVQUAL model, which has limitations for assessing the satisfaction of digital innovations. However, the stability of the scale and sufficient convergent validity make the model appropriate for this study.

## 4 Results

**Fig. 2:**
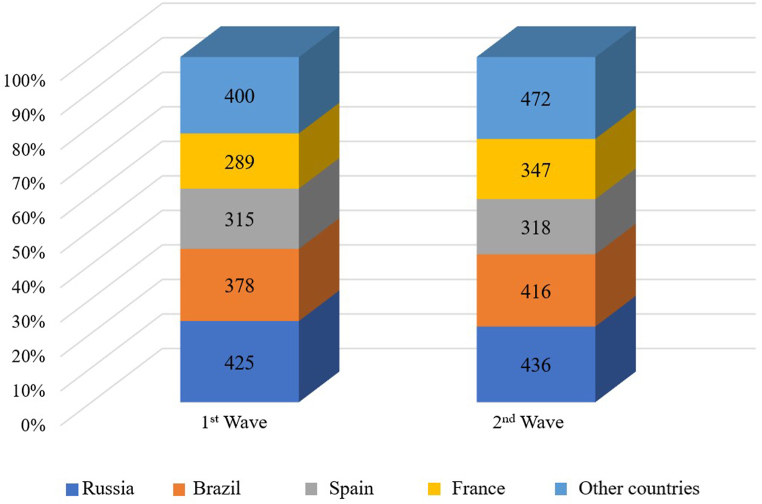
Respondent distribution by country of origin.

In the first phase of the study, the participants represented 67 countries, while the second phase covered respondents from 73 countries worldwide. Due to the substantial number of countries, the respondent distribution by country of origin is presented in aggregate form for the first and second phases of the study. Thus, Fig. 2 shows countries providing at least 2/3 of the respondents, with others grouped as “other countries”.

**Fig. 3:**
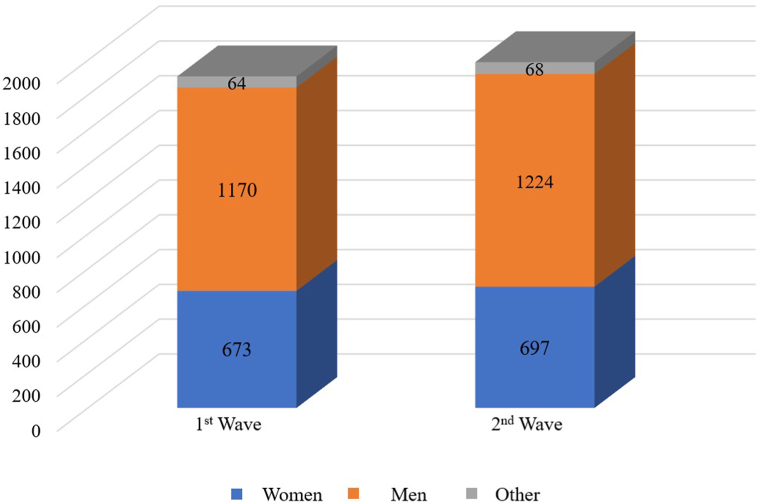
Gender structure of respondents.

The leading countries in terms of the number of purchases in both the first and second phases of the study were Russia, Brazil, Spain, and France. Respondents from these countries accounted for approximately 78 % in the first phase and 75 % in the second phase of the study. The total number of countries making purchases on AliExpress and data on countries leading in the number of purchases [57] align with the structure of buyers on the trading platform. This indirectly confirms the representativeness of the sample. The gender structure of the respondents is presented in Fig. 3.

**Fig. 4:**
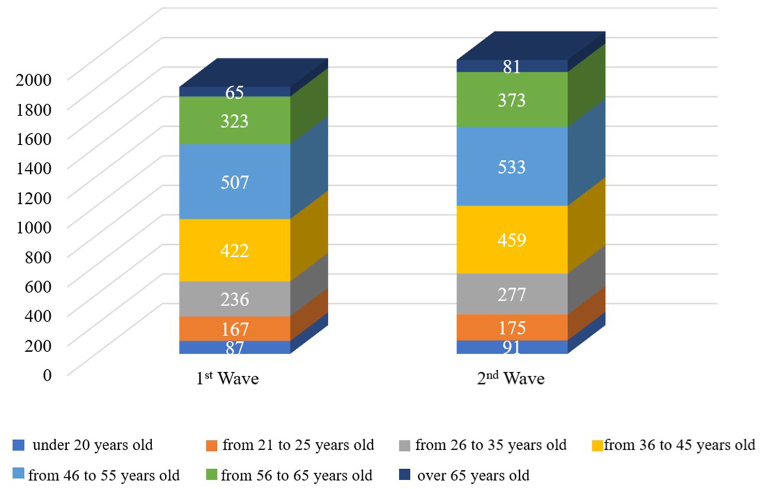
Age structure of respondents.

Among the respondents, the proportion of men was higher (61 % in the first phase and 62 % in the second phase). The gender structure also corresponds to the gender structure of AliExpress platform buyers [57]. Again, this data indirectly supports the representativeness of the sample. The age structure of the respondents is presented in Fig. 4.

**Fig. 5:**
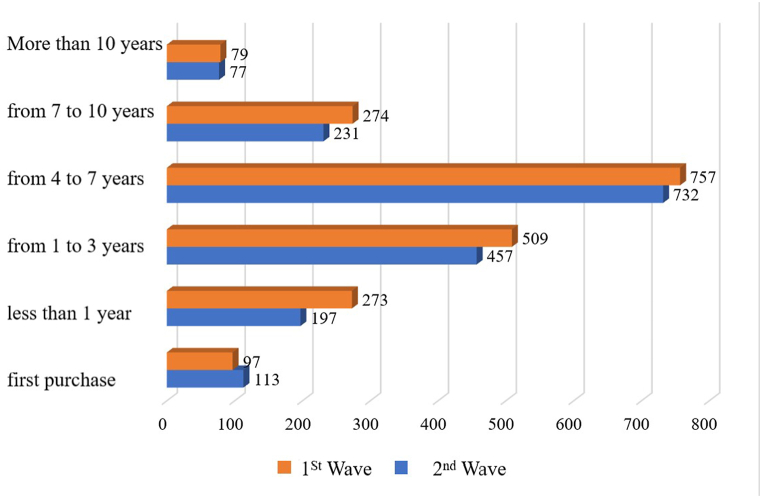
Respondents' experience with international purchases.

The age structure of respondents was balanced and relatively stable. Among the respondents, the majority were within the age range of 36–45 years (23 % in both the first and second phases) and 46–55 years (28 % in the first phase and 27 % in the second phase). Overall, half of the respondents in both the first and second phases of the study are older than 45 years, aligning with the age profile of buyers on the AliExpress trading platform [57]. This also indirectly confirms the representativeness of the sample. Fig. 5 presents data on respondents' experience with international purchases.

At the time of the survey, the majority of respondents (41 % in the first phase and 38 % in the second phase) had been making international purchases for 4–7 years. Another 1/4 of respondents (25 % in the first phase, 26 % in the second phase) had been engaging in international purchases for 1–3 years. Approximately 5 % of respondents (5 % in the first phase, 6 % in the second phase) made their first international purchase. Thus, expectations regarding international purchases were based on the knowledge of reference groups and other borrowed judgments for only 5 % of respondents. In turn, 95 % of respondents formed their expectations based on their own previous experience. Table 2 presents the analysis of the acceptability, normality, and reliability of the sample for the first phase of the study.

**Table 2:** Analysis of sample acceptability, normality, and reliability (first phase).

Feedback statements	Average Value	Standard Deviation	Item–total correlation
My order has been delivered on time.	4.33	0.71	0.69
The item I received matches the description.	4.47	0.58	0.49
The quality of the item exceeded my expectations.	3.95	0.84	0.69
The process of selecting and ordering items on the website was intuitive.	4.47	0.54	0.49
The packaging of the order was neat and ensured the safety of transportation.	4.43	0.69	0.46
The delivery was fast from the moment of placing the order to receiving it.	4.22	0.80	0.76
I received timely notifications about the status of my order.	4.32	0.63	0.54
I had no doubts about the security of my data during the purchase.	4.36	0.59	0.44
I felt that the seller cared about my comfort during the purchase.	3.97	0.84	0.68

As observed, the value of the item-total correlation coefficient for all questions exceeds 0.4. This result indicates a high level of correlation and confirms the acceptability, normality, and reliability of the sample. A subsequent analysis was conducted to study the acceptability, normality, and reliability of the sample for assessing customer satisfaction among those who contacted customer support (Table 3).

**Table 3:** Analysis of acceptability, normality, and reliability of the sample for customer satisfaction among those contacting customer support (first phase).

Feedback statements	Average Value	Standard Deviation	item–total correlation
I was provided with the necessary information about the products when I requested it.	4.38	0.63	0.71
The customer support responds quickly to my inquiries.	3.94	0.84	0.77
When I encountered issues with my order, the staff was ready to help.	4.46	0.57	0.71

As the table shows, the item-total correlation coefficient for all questions is above 0.7. This result indicates a very high level of correlation and confirming the acceptability, normality, and reliability of the sample of customers who contacted customer support. The number of respondents confirms the representativeness of this sample for the entire respondent population. Therefore, it is possible to group the obtained data according to the key service quality parameters within the SERVQUAL model, which provides an integrated indicator of customer satisfaction (Table 4).

**Table 4:** Customer satisfaction across key dimensions of service quality in the SERVQUAL model (first phase).

Parameters	Indicator
**Reliability**	**4.25**
My order has been delivered on time	4.33
The product I received matches the description	4.47
The quality of the product exceeded my expectations	3.95
**Responsiveness**	**4.16**
I was provided with the necessary information about the products when I requested it.	4.38
Customer support promptly responds to my inquiries.	3.94
**Completeness**	**4.37**
The process of selecting and ordering products on the website was intuitively understandable.	4.47
The packaging of the order was neat and ensured the safety of transportation.	4.43
The delivery was fast from the moment of placing the order to receiving it.	4.22
**Assurance**	**4.34**
I received timely notifications about the status of my order.	4.32
I had no doubts about the security of my data during the purchase.	4.36
**Empathy**	**4.22**
I felt that the seller cared about my comfort during the purchase.	3.97
When I had issues with the order, the staff was ready to help.	4.46
**Integrated Indicator**	**4.27**

According to the results of the first phase, the level of satisfaction with the purchase was high – 4.23. The highest score was recorded for the “Completeness” dimension - 4.37, while the lowest score was for the “Responsiveness” dimension – 4.16. The implementation of digital innovations involved the integration of additional digital features on the platform, including functions such as Fast Shipping and Combined Fast Shipping. Additionally, within the project, a specific innovation was introduced – a chatbot that provided instant customer support by selected stores. The implementation of these innovations was followed by a second phase of the research (Table 5).

**Table 5:** Customer satisfaction across key dimensions of service quality in the SERVQUAL model (second phase).

Parameters	Indicator
**Reliability**	**4.37**
My order has been delivered on time.	4.63
The product I received corresponds to the description.	4.49
The quality of the product exceeded my expectations.	3.98
**Responsiveness**	**4.01**
I was provided with the necessary information about the products when I requested it.	3.99
Customer support responds quickly to my inquiries.	4.03
**Completeness**	**4.59**
The process of choosing and ordering products on the website was intuitive.	4.52
The packaging of the order was neat and ensured the safety of transportation.	4.51
Delivery was fast from the moment of ordering to receiving the order.	4.73
**Confidence**	**4.56**
I received timely notifications about the status of my order.	4.6
I had no doubts about the security of data during the purchase.	4.51
**Empathy**	**3.88**
I felt that the seller cared about my comfort during the purchase.	3.89
When I had problems with the order, the staff was ready to help.	3.87
**Integrated Indicator**	**4.28**

**Fig. 6:**
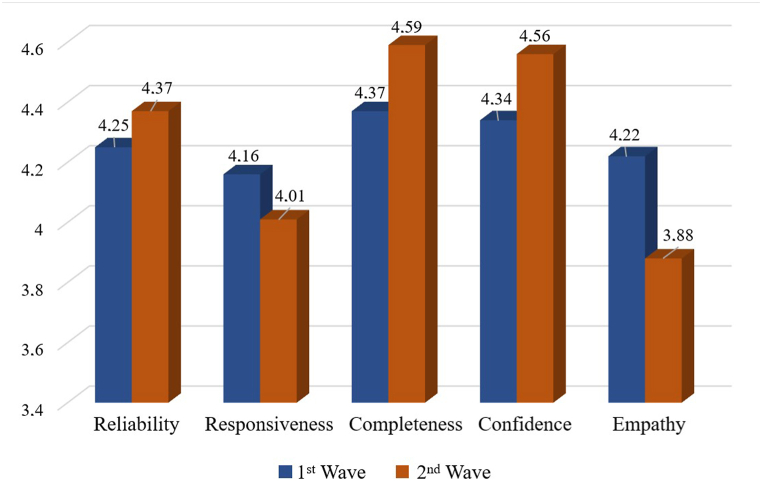
Dynamics of changes in customer satisfaction as a result of implementing digital innovations.

As evidenced, digital innovations led to substantial changes in customer satisfaction across nearly all dimensions of the SERVQUAL model. The integral satisfaction indicator showed some improvement. There was also an increase in positive feedback regarding the reliability of order delivery, with indicators such as delivery time, accuracy of the provided description and product quality exceeding customer expectations. Additionally, the study revealed an increase in positive feedback related to completeness (intuitive process of ordering, reliable packaging, and fast delivery) among AliExpress customers. This confirms hypothesis H1 of this study, suggesting that digitization may significantly impact the level of customer satisfaction in international trade under post-COVID economic conditions. Fig. 6 illustrates the dynamics of changes in customer satisfaction.

Thus, the implementation of basic digital innovations has significantly enhanced indicators in several dimensions. The perception of reliability has improved by 0.12 compared to the baseline, primarily due to the increase in the assessment of timely delivery (+0.3). At the same time, the slight increase in the indicators of description accuracy (+0.02) and product quality assessment (+0.03) can be explained by a positive impact of faster and more reliable delivery. However, the growth of these indicators may be a subject for further research to obtain additional empirical confirmations.

The perception of completeness has improved by 0.22 compared to the baseline, primarily due to the enhancement in order processing and delivery speed (+0.51). Additionally, within this dimension, customer satisfaction with product packaging (+0.08) and the process of selecting and ordering items (+0.05) have improved. There was also an increase by 0.22 points compared to the baseline in customers' confidence in purchases, mainly due to increased satisfaction with the regularity of notifications (+0.28) and an enhanced perception of data security (+0.15). However, the implementation of a chatbot to ensure fast customer support only increased satisfaction with the support response speed (+0.09), while satisfaction with providing additional information significantly decreased (−0.39). As a result, the indicators for the “Responsiveness” dimension decreased by 0.15 points.

Furthermore, the introduction of the chatbot resulted in a significant decrease in Empathy (−0.34), primarily due to a sharp decline in the staff's readiness to help (−0.59) and a reduction in satisfaction with customer support (−0.08). Thus, hypothesis H2 has received empirical confirmation that individual elements of digitization can have both positive and negative effects on customer satisfaction in the post-COVID economy.

The projected change in customer satisfaction upon cancelation of the chatbot is presented in Table 6.

**Table 6:** Projected changes in customer satisfaction upon cancellation of the chatbot.

	First Phase	Second Phase	Projected Number
Reliability	4.25	4.37	4.37
Responsiveness	4.16	4.01	4.16
Completeness	4.37	4.59	4.59
Confidence	4.34	4.56	4.56
Empathy	4.22	3.88	4.22
Integrated Indicator	4.27	4.28	4.38

Thus, the projected changes in AliExpress customer satisfaction according to certain criteria show an increase in terms of reliability, completeness, and confidence. However, there is a slight drop in Responsiveness and Empathy between the first and second phases. In general, the analysis of the projected changes in customer satisfaction showed that the cancellation of the chatbot would increase customer satisfaction by 0.11 (compared to 0.01 with the introduction of all innovations). This indicates the expediency of canceling this digital innovation on the platform. In conclusion, it should be noted that the study demonstrated the significant influence of digitization on customer satisfaction levels in international trade. The specific elements of digitization were found to have both positive and negative impacts on customer satisfaction under economic conditions post-COVID.

## 5 Discussion

This study examined the interrelation and mutual influence of digitization in international trade on customer satisfaction in the post-COVID economic environment. The study used the SERVQUAL model to assess the quality of service and compare customer perceptions and expectations by several key parameters (through the survey). The investigation revealed gaps in the provision of services. The findings allowed the authors to develop recommendations for improving the international online retail experience on AliExpress. The developed questionnaire meets the accepted estimates of validity and reliability. Nevertheless, the current research necessitates a more critical approach to the justification and use of estimates on a multi-point scale [58]. To solve possible problems of representativeness, tools such as CLC Estimator can be utilized [59].

Hypothesis H1 has been confirmed, suggesting that digitalization can have a statistically significant impact on customer satisfaction in international trade in the economic conditions after COVID. The introduction of digital innovations led to significant changes in almost all dimensions of the SERVQUAL model and an increase in the integral indicator of AliExpress customer satisfaction. Thus, the findings support the conclusions presented in earlier scientific papers, stating that customer satisfaction largely depends on the strategy for implementing digital service tools [60]. Previous studies have assumed that customer satisfaction in the digital environment increases the efficiency of the online trading business [61]. Some authors have suggested that the older generations of customers experience less satisfaction from the introduction of digital purchases and payments [62]. Nevertheless, this study demonstrated that the impact of digitalization was the same for the whole population.

Hypothesis H2 has also been confirmed. The findings show that certain elements of digitalization can have both positive and negative effects on customer satisfaction in the post-pandemic economy. In this case, the introduction of a chatbot led to a significant decrease in Empathy. This change is attributed to a sharp decline in the staff's readiness to help and a reduction in satisfaction with customer support. In general, this study confirms the conclusions of previous research that aspects of service quality can negatively impact customer satisfaction, even in a highly digitized business environment [63]. However, in some cases, the effect of "fluke” may have a decisive role [64]. An unexpected positive experience of using digital innovations can have an additional impact on the overall satisfaction of customers.

This study also demonstrated that the implementation of basic digital innovations improved the perception of reliability. The use of innovative technologies enhanced customer satisfaction with order processing speed and delivery, packaging of products, and the process of selecting and ordering items, as well as the regularity of notifications and data security. Some of these aspects were also validated in studies related to customer service. For instance, in the healthcare sector, digital technologies increased satisfaction with the speed of addressing inquiries [65]. Research has also demonstrated that the simplicity of using digital technologies positively impacts perceived confidentiality, which, in turn, positively affects the perceived security by the customer [66]. However, the implementation of chatbots was shown to deteriorate customer-perceived Responsiveness and cause a significant decrease in Empathy. This finding supports previous studies, which asserted that the introduction of artificial intelligence in customer interactions may result in not only benefits, but also risks for businesses. Not all customers positively perceive this type of communication [67].

## 6 Conclusions

The results of the study confirmed the impact of digitalization on customer satisfaction in international trade operations. After the introduction of digital innovations, the level of customer satisfaction has changed in almost all SERVQUAL dimensions. This change serves as an empirical confirmation of hypothesis H1. There were significant improvements in terms of perceived reliability and satisfaction with order processing and delivery speed, packaging, the process of ordering, the regularity of notifications, and the perception of data security. Nevertheless, certain elements of digitalization may result in a decrease in customer satisfaction (empirical support for hypothesis H2 has been provided). The introduction of a chatbot reduced satisfaction with the provision of additional information, which negatively affected the indicators of Responsiveness and Empathy. The findings suggest that it may be advisable to cancel the use of chatbots for providing services to the customers of online stores.

### Limitations and future research directions

The selection of stores for the study was random. However, certain criteria were used to ensure the possibility of implementing additional platform features. Based on these criteria, the study involved only one international trading platform (AliExpress). This limitation may be addressed in subsequent research. The participation of store customers in the study was voluntary, but their involvement was incentivized. Incentivizing participation in the survey was achieved through targeted personal discounts (coupons) for the next purchase. This approach could have attracted the most motivated customers from the platform's sample to participate in the survey. When adding an item to the cart, the buyers received a notification about the amount they could save by participating in the survey. After making the purchase and payment, the buyers were invited to fill out an online survey form. The submission of the completed questionnaire activated the coupon for the next purchase. Overall, this measure did not statistically affect the overall random nature of the sample of survey participants.

Possible directions for future research on customer satisfaction in the context of digitalization include the use of similar approaches to test the model. This could provide more accurate data on the studied variables. It would also be beneficial to scrutinize the effect of "fluke” to assess how customer satisfaction changes if the experience of using digital innovations becomes unexpectedly positive for them. In this context, it is necessary to focus on fundamentally new digital innovations that customers have not encountered in their previous shopping experiences on online platforms.

### Research implications

The academic contribution of the findings complements existing research on the mechanism of enhancing customer perception of specific innovations. The study presents valuable insights, structuring and expanding scientific data on the impact of digitization on customer satisfaction in international trade in the post-COVID-19 period. The practical outcome of this study lies in the empirical confirmation of the need to test customer satisfaction when implementing digital innovations. According to the results, not all digital innovations are perceived equally positively by customers and are able to increase their satisfaction. Therefore, practitioners of international digital trading platforms are recommended to refine the use of certain tools, such as chatbots, in order to improve their effectiveness. The implementation of such tools requires a tailored approach that aims to preserve the benefits of innovations while ensuring increased customer satisfaction.

## Funding

This research was supported by the "National First Class Undergraduate Course in Internet Marketing".

## Data availability

Data will be available upon request.

## Ethics approval

The research was conducted ethically in accordance with the World Medical Association Declaration of Helsinki. The research was approved by the local ethics committees of Qingdao Huanghai University (Protocol no. 4993 dated February 02, 2023).

## Informed consent

Informed consent was signed by the participants.

## CRediT authorship contribution statement

**Guangying Wang:** Validation, Software, Methodology, Formal analysis, Conceptualization.

## Declaration of competing interest

The authors declare that they have no known competing financial interests or personal relationships that could have appeared to influence the work reported in this paper.

## Appendix A

### Questionnaire

1. Please specify the country where you placed your order from: _______________________________________
2. Your gender:
  Female □
  Male □
  Other □
3. Your age:
  Younger than 20 years □
  21–25 years □
  26–35 years □
  36–45 years □
  46–55 years □
  56–65 years □
  Older than 65 years □
4. Your experience with international purchases:
  First purchase □
  Less than 1 year □
  1–3 years □
  4–7 years □
  7–10 years □
  More than 10 years □
5. Evaluate your agreement or disagreement with the statements using the following rating scale:
  Strongly agree - 5
  Somewhat agree, somewhat disagree - 4
  Neutral - 3
  Somewhat disagree, somewhat agree - 2
  Strongly disagree - 1
    5.1.1 My order has been delivered on time □
    5.1.2 The product I received matches the description □
    5.1.3 The quality of the product exceeded my expectations □
    5.2.1 Customer support responds quickly to my requests □
    5.2.2 I was provided with the necessary information about products when I asked □
    5.3.1 The process of selecting and ordering products on the website was intuitive □
    5.3.2 The packaging of the order was neat and ensured safe transportation □
    5.3.3 The delivery was fast from the time of placing the order to receiving it □
    5.4.1 I received timely notifications about the status of my order □
    5.4.2 I had no doubts about the security of my data during the purchase process □
    5.5.1 I felt that the seller cared about my comfort during the purchase □
    5.5.2 When I encountered problems with the order, the staff was ready to help □
